# Recent Advances in the Synthesis of Di- and Trisubstituted Hydroxylamines

**DOI:** 10.3390/molecules28062816

**Published:** 2023-03-21

**Authors:** Jarvis Hill, Thomas D. Beckler, David Crich

**Affiliations:** 1 Department of Pharmaceutical and Biomedical Sciences, University of Georgia, 250 West Green Street, Athens, GA 30602, USA; 2 Department of Chemistry, University of Georgia, 302 East Campus Road, Athens, GA 30602, USA; 3 Complex Carbohydrate Research Center, University of Georgia, 315 Riverbend Road, Athens, GA 30602, USA

**Keywords:** synthesis, hydroxylamines, chemical reactions, amines, chemical space, heteroatoms, electrophiles

## Abstract

As an underrepresented functional group in bioorganic and medicinal chemistry, the hydroxylamine unit has historically received little attention from the synthetic community. Recent developments, however, suggest that hydroxylamines may have broader applications such that a review covering recent developments in the synthesis of this functional group is timely. With this in mind, this review primarily covers developments in the past 15 years in the preparation of di- and trisubstituted hydroxylamines. The mechanism of the reactions and key features and shortcomings are discussed throughout the review.

## 1. Introduction

**Figure 1:**
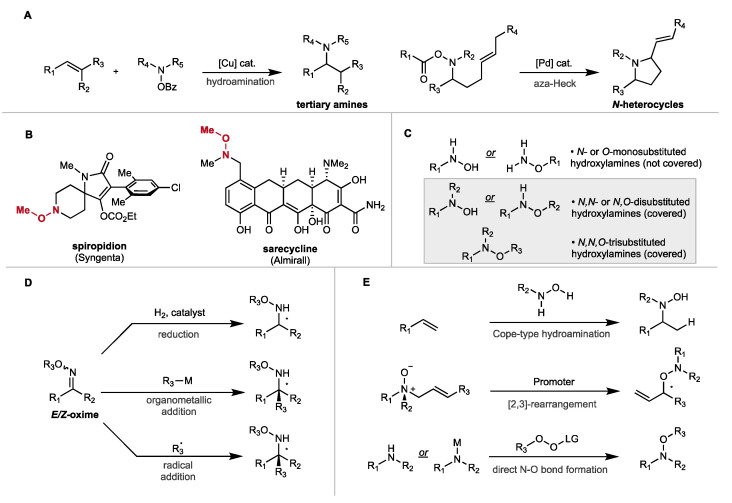
(**A**) Examples of the use of *O*-acyl-*N*,*N*-disubstituted hydroxylamines in synthesis. (**B**) The *O*-methoxy-*N*,*N*-disubstituted hydroxylamine moiety in approved agrochemical and pharmaceutical drug structures. (**C**) Hydroxylamine terminology and scope of the present review. (**D**) Examples of oxime transformations toward disubstituted hydroxylamines. (**E**) Select examples of hydroxylamine synthesis covered in this review.

In synthetic chemistry, the *O*-acyl-*N*,*N*-disubstituted hydroxylamine moiety has received a great deal of attention on account of its ability to function as an electrophilic nitrogen source [1,2,3,4]. Indeed, this mode of reactivity has proved to be highly effective in the synthesis of functionalized chiral tertiary amines through copper-catalyzed hydroamination procedures, or of chiral *N*-heterocycles through palladium-catalyzed aza-Heck/aza-Narasaka-Heck cyclizations as popularized by many groups, notably those of Buchwald and Bower, respectively (Figure 1A) [5,6,7,8]. Conversely in nature, the analogous *O*-acetylation or *O*-sulfonylation of hydroxylamines, followed by elimination to nitroso derivatives, is called phase II metabolism of hydroxylamines and is the root cause of hydroxylamine-based mutagenicity [9,10]. Compared to *O*-acyl-*N*,*N*-disubstituted hydroxylamines and related derivatives, the chemistry of di- and trialkylhydroxylamines is much less studied. With experimentally determined bond dissociation energy (BDE) values from 55 to 65 kcal·mol^−1^ a pKa of 5.93 in aqueous solution for the conjugate acid of hydroxylamine itself, and barriers to stereomutation, nitrogen inversion, or N-O bond rotation of approximately 15 kcal·mol^−1^, the di- and trialkylhydroxylamine units offer interesting properties situated in unique chemical space [11,12,13,14,15,16]. The trialkylhydroxylamine unit is found in approved scaffolds such as the anti-insecticidal spiropidion, developed by Syngenta, the tetracycline-derived antibiotic sarecycline, developed by Allergen (acquired by Almirall), and has been used as a phosphate replacement in nucleotide analogs by Alnylam, but such examples are rare (Figure 1B) [17,18,19]. Despite these examples, broader uptake of the hydroxylamine unit in small-molecule drug discovery settings beyond simple *O*-methyl-*N*,*N*-disubstituted hydroxylamines has yet to materialize. This could be due in part to the assignment of hydroxylamines as “structural alerts” in traditional medicinal chemistry or to the historical lack of reliable synthetic methods for this functionality [20,21].

The aim of this review is to summarize recent developments in synthetic methodology directed toward the preparation of the di- and trialkylhydroxylamine moieties (Figure 1C). We begin by discussing approaches to dialkylhydroxylamines through catalytic reduction of oxime ethers, then continue with some recent advances in alkylation and reductive amination chemistry for the preparation of di- and trialkylhydroxylamines. Then, we move into recent developments in Cope-type hydroaminations and [2,3]-Meisenheimer rearrangements of *N*-oxides and finally conclude with developments in di- and trialkylhydroxylamine synthesis by direct N-O bond formation (Figure 1D,E). Emphasis is placed on developments made in the past 15 years for synthetic methods aimed at acyclic di- and trialkylhydroxylamines: for a more thorough discussion on the chemistry of electrophilic hydroxylamine derived reagents [3], hydroxamic acids [22], oximes [23], or the chemistry of *endo*-cyclic hydroxylamines [24], the reader is directed to some recent reviews.

## 2. Synthesis of Disubstituted Hydroxylamines by Catalytic Reduction of Oxime Ethers

**Scheme 1:**
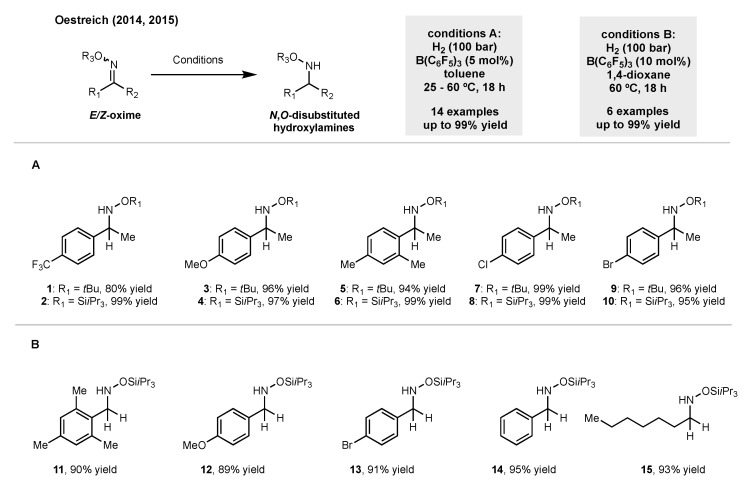
Oestreich’s hydrogenation of *E*/*Z* oximes using frustrated Lewis pairs. (**A**) Selected examples of the reaction on ketoximes using conditions A. (**B**) Selected examples of the reaction on aldoximes using conditions B [32,33].

The reduction of oxime ethers is a valuable method for the synthesis of N,O-disubstituted hydroxylamines with high step- and atom-economy [25]. Unfortunately, traditional methods are largely based on reduction with stoichiometric borohydrides [26,27], hydrosilanes [28], or organotin hydrides [29]. Moreover, oxime ether reduction is complicated by the requirement for selective reduction of the C=N bond without reductive cleavage of the labile N-O bond [25]. Since Vavon’s seminal reports in the 1920s on the first catalytic reduction of oxime ethers, much effort has been directed toward improving yields, functional group tolerance, and in the development of stereoselective variants, most notably using homogenous catalysts [30,31]. Thus, in 2014, Oestreich reported a frustrated Lewis pair-catalyzed hydrogenation of sterically encumbered ketoxime derivatives using a tris(pentafluorophenyl)borane catalyst in toluene at 100 bar of hydrogen pressure (Scheme 1) [32,33]. Under the optimized conditions, excellent yields (up to 99%) were obtained for the *N*,*O*-disubstituted hydroxylamines and a variety of functional groups could be tolerated including trifluoromethyl (**1**–**2**) in 80–99% yields, and aryl halides with products (**7**–**10**) isolated in 95–99% yield (Scheme 1A). However, when the optimized conditions were applied to aldoxime substrates no reaction occurred, which the authors attributed to the reduced Lewis basicity of aldoximes relative to ketoximes. To enable efficient reduction of aldoxime substrates, the authors switched the solvent from toluene to 1,4-dioxane, which was precedented to act as the Lewis-basic component in frustrated-Lewis-pair-type heterolytic dihydrogen splitting [33,34,35]. Under these conditions, various aldoximes underwent efficient reduction of the C=N bond selectively to afford *N*,*O*-disubstituted hydroxylamines (**11**–**15**) in excellent yields (89–95%) (Scheme 1B).

**Scheme 2:**
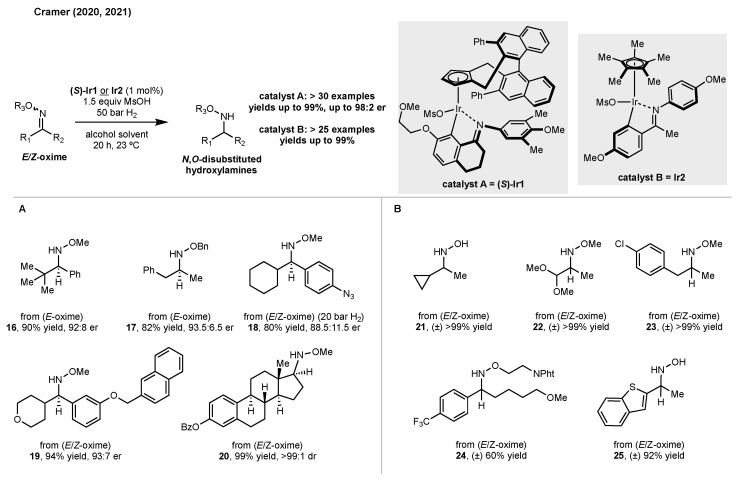
Cramer’s enantioselective and racemic hydrogenations of *E*/*Z* oximes for the synthesis of secondary hydroxylamines. (**A**) Selected examples of the enantioselective reaction using catalyst [(*S*)-Ir1]. (**B**) Selected examples of the racemic reaction using catalyst [Ir2] [36,37].

In 2020, Cramer reported the first highly efficient stereoselective reduction of oximes using chiral cyclometalated Cp Ir(III) methanesulfonate complexes ((*S*)-Ir1) in an alcohol solvent with 1.5 equivalents of methanesulfonic acid under 50 bar of hydrogen pressure at room temperature (Scheme 2) [36]. Importantly, the reaction proceeded without the requirement for a bulky substituent as seen in Oestrich’s frustrated-Lewis-pair reduction protocol, while retaining full chemoselectivity towards the oxime C=N bond with catalyst turnovers up to 4000 and enantiomeric ratios up to 98:2 [33,35]. Under the optimized conditions, a variety of substrates underwent efficient reduction even with sterically congested oximes such as a *tert*-butyl substituted oxime that gave the intended product (**16**) in 90% yield (Scheme 2A). A variety of functional groups were well tolerated including ethers and esters affording products (**19**–**20**) in excellent yields (94–99%) (Scheme 2A). Using a slightly modified version of the standard reaction conditions with hydrogen pressure reduced to 20 bar, even azide-bearing oxime ethers were competent substrates in the transformation affording the intended products (**18**) in 80% yield (Scheme 2A). In a subsequent 2021 study by Cramer aimed at investigating the full scope and reaction mechanism of the novel hydrogenation procedure, an alternative cyclometalated Cp* Ir(III) methanesulfonate complex (Ir2) was identified as a highly efficient catalyst for hydrogenation [37]. Using the new catalyst, selective C=N reduction of oximes containing broad functionalities could be achieved in up to 99% yield with examples including cyclopropyl (**21**), dimethylacetal (**22**), phthalimido (**24**), or benzothiazole (**25**) carrying oximes (Scheme 2B).

**Scheme 3:**
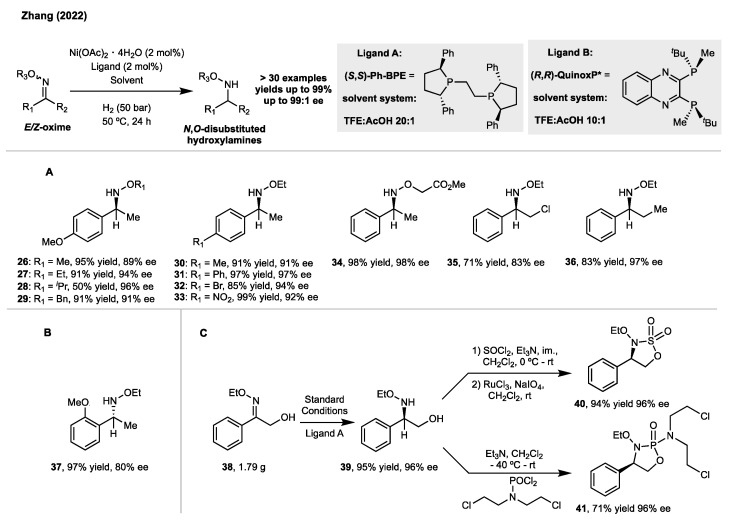
Zhang’s stereoselective hydrogenation of *E*/*Z* oximes using nickel catalysis. (**A**) Selected examples of the transformation using ligand system A. (**B**) Example of the transformation with an *ortho*-bearing substrate using ligand system B. (**C**) Gram-scale transformation and subsequent derivatization [38].

In 2022, Zhang reported the first asymmetric reduction of oximes using earth-abundant Ni catalysis. This reaction proceeds under 50 bar hydrogen pressure with the addition of acid in up to 99% yield and 99% e.e. of the product *N*,*O*-disubstituted hydroxylamines (Scheme 3) [38]. Under the optimized condition, ketoximes were reduced in excellent yields (up to 99%) to the corresponding *N*,*O*-disubstituted hydroxylamines (**26**–**36**) (Scheme 3A). A variety of functional groups were well tolerated in the reaction including aryl halides and nitro groups, with products (**32**, **33**) formed in excellent yields (85% and 99%, respectively). Moreover, a carbomethoxy substituted oxime ether functioned well as a substrate affording the intended *N*,*O*-disubstituted hydroxylamine (**34**) in 98% yield while retaining excellent enantioselectivity (98:2). For *ortho*-substituted aryl ketoximes a variation in reaction conditions was needed to affect reduction, with a Ni/(*R*,*R*)-Quinoxp* catalyst being optimal under slightly adjusted solvent conditions (TFE/AcOH = 10:1) giving an *N*,*O*-disubstituted hydroxylamine (**37**) in 97% yield (Scheme 3B). A gram scale reduction was also carried out on (**38**) with a substrate-to-catalyst ratio of 1000:1, affording the intended *N*,*O*-disubstituted hydroxylamine (**39**) in 95% yield and excellent enantioselectivity (98:2). To highlight the potential synthetic utility of the transformation, hydroxylamine (**39**) was also converted into the *N*-ethoxy oxathiazolidine (**40**) and the cyclophosphamide (**41**) in 94% and 71% yields, respectively (Scheme 3C).

## 3. Protecting Group-Free Synthesis of Di- and Trisubstituted Hydroxylamines

Historically, alkylation chemistry represented the most common route for the preparation of di- and trisubstituted hydroxylamines [39]. This is likely due to the widespread availability of *N*-hydroxyphthalimide (NHPI) and hydroxylamine hydrochloride, which provide reliable methods for the preparation of mono- and disubstituted hydroxylamines but that nevertheless require a series of protection/deprotection steps to achieve the intended products [39]. Synthesis of hydroxylamines by alkylation chemistry, however, is limited to the use of strong electrophiles, which in turn leads to complications due to overalkylation. For example, selective mono-alkylation of *O*-monosubstituted hydroxylamines is fraught with challenges due to competing dialkylation in comparison to the more straightforward alkylation of *N*,*O*-disubstituted hydroxylamines [39,40]. In this section, we will cover recent strategies for the direct preparation of di- and trisubstituted hydroxylamines from less substituted hydroxylamines without protection/deprotection sequences.

**Scheme 4:**
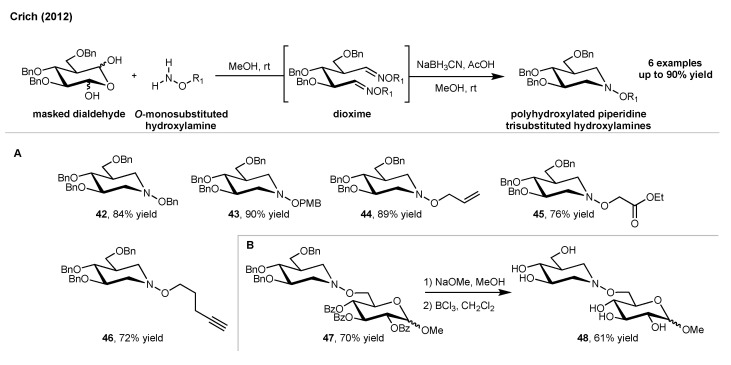
Crich’s double reductive amination procedure for the synthesis of polyhydroxylated piperidine-derived trisubstituted hydroxylamines. (**A**) Selected examples of the transformation. (**B**) Application to complex hydroxylamines and deprotection [44].

In contrast to alkylation chemistry, reductive amination procedures of *O*-monosubstituted hydroxylamines represent a powerful strategy for the synthesis of *N*,*O*-disubstituted and trisubstituted hydroxylamines [41]. This is underlined by the commercial preparation of sarecycline (Figure 1B) by Almirall, which employs a reductive amination between *N*,*O*-dimethylhydroxylamine and a tetracycline-derived aldehyde followed by reduction with borane to construct the trisubstituted hydroxylamine moiety [42]. Beyond simple *O*-methoxy-*N*,*N*-disubstituted hydroxylamines, in 1994, Bols reported a concise synthesis of isofagomine which included a ring-closing double reductive amination step for the construction of the polyhydroxylated piperidine ring [43]. In 2012, Crich applied a related strategy for the synthesis of polyhydroxylated *N*-alkoxy piperidines (Scheme 4) [44]. Under the optimized conditions, which consisted of dioxime formation from a masked dialdehyde, followed by reductive ring-closure, a variety of trisubstituted hydroxylamines could be prepared bearing a range of functional groups, including benzyl (**42**), paramethoxybenzyl (**43**), allyl groups (**44**), ethyl esters (**45**), and alkynes (**46**) (Scheme 4A). Moreover, the reaction could be applied to the synthesis of complex disaccharide mimetics linked through a trisubstituted hydroxylamine, where the intended product (**47**) was isolated in 70% yield (Scheme 4B). Disaccharide (**47**) could then be deprotected under Zemplén conditions (sodium methoxide in MeOH) followed by BCl_3_-mediated cleavage of the benzyl ethers, giving the intended product (**48**) in 61% yield over two steps, all while retaining the hydroxylamino linkage [45]. This ring-closing double reductive amination approach was also later used by Crich in the synthesis of di- and trimeric hydroxylamine based β-(1→3)-glucan mimetics [46,47].

**Scheme 5:**
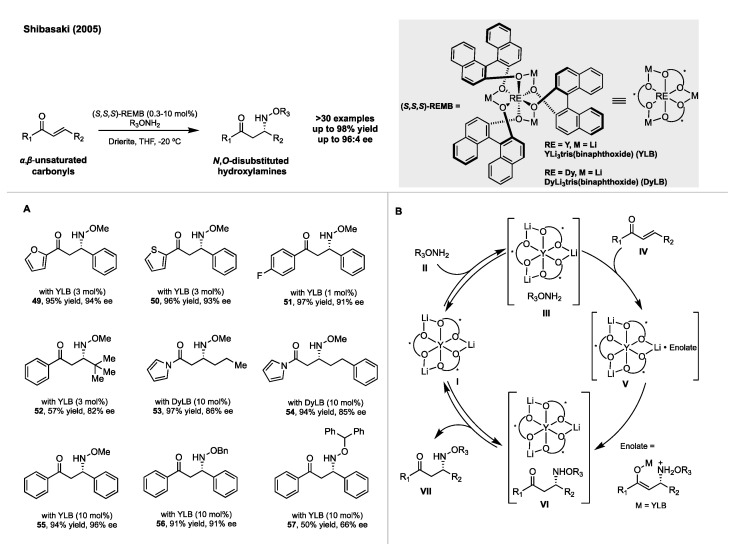
Shibasaki’s rare-earth-metal-BINOL (REMB) catalyzed enantioselective conjugate addition of hydroxylamines to *α,β*-unsaturated carbonyls. (**A**) Selected examples of the transformation. (**B**) Proposed catalytic cycle [48,49,50,51].

Shibasaki’s rare earth alkali metal BINOLate (REMB) framework has found wide applications in the preparation of enantioenriched small molecules through simple modification of the pro-ligand, (*R*)- or (*S*)-BINOL [48,49,50,51]. In the early 2000s, Shibasaki reported that the REMB framework could be used in the synthesis of highly enantioenriched *N*,*O*-disubstituted hydroxylamines by conjugate addition of *O*-alkylhydroxylamines to α,β-unsaturated carbonyl derivatives in up to 98% yield and 96% e.e. (Scheme 5) [50]. Using yttrium-based (*S*,*S*,*S*)-REMB (YLB), high yields (up to 97%) and excellent enantioselectivities (up to 96:4) were obtained of the *N*,*O*-disubstituted hydroxylamines with good functional group tolerance of the enone substrates, which included furans (**49**), thiophenes (**50**), and aryl halides (**51**) (Scheme 5A). However, in the conjugate addition of *O*-methylhydroxylamine to α,β-unsaturated *N*-acylpyrroles the authors noted that a dysprosium-based REMB (DyLB) gave slightly better yields and enantioselectivities (up to 97% yield, 86:14 e.e.) than the original YLB catalyst. The reaction was also extended to homologs of *O*-methoxy disubstituted hydroxylamine, with *O*-benzylhydroxylamine and *O*-diphenylmethylhydroxylamine affording products (**56**, **57**) in 91% and 50% yield, respectively. The reduced yield of **57** was accounted for by the sterically encumbered nature of the nucleophile, which the authors claimed highlighted the importance of complexation between YLB and the hydroxylamine nucleophile on the outcome of the reaction. The authors also proposed a catalytic cycle that begins with the reversible coordination of hydroxylamine (**II**) to the YLB catalyst (**I**) to form an active YLB-hydroxylamine catalyst (**III**) (Scheme 5B). Upon reaction of **III** with the α,β-unsaturated carbonyls (**IV**) in a carbon–nitrogen bond forming event, enolate (**V**) is formed and then undergoes irreversible proton transfer to yield **VI**. Upon dissociation, the intended product (**VII**) is formed, and active YLB-hydroxylamine catalyst (**III**) is regenerated after another coordination event with hydroxylamine (**II**). The importance of conjugate additions of lesser substituted hydroxylamines in the construction of di- and trisubstituted hydroxylamines is evident from the synthesis of the anti-insecticidal, spiropidion (Figure 1B): a double conjugate addition of *O*-methylhydroxylamine with two molecules of methyl acrylate is followed by Dieckmann reaction to afford the requisite intermediate trisubstituted hydroxylamine, *N*-methoxy-4-piperidinone [17,52].

**Scheme 6:**
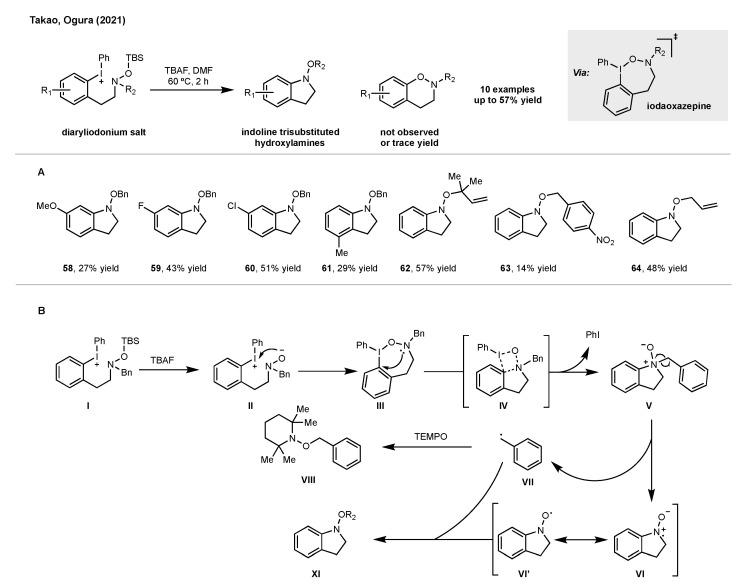
Takao and Ogura’s synthesis of indoline trisubstituted hydroxylamines. (**A**) Selected examples of the transformation. (**B**) Proposed mechanism [53].

In 2021, Takao and Ogura reported a synthesis of indoline-based trisubstituted hydroxylamines by reaction of diaryliodonium salts with tetrabutylammonium fluoride (TBAF) through a proposed iodaoxazepine intermediate (Scheme 6) [53]. Under these conditions, a variety of indoline-based trisubstituted hydroxylamines could be obtained in fair yields (up to 57%) with good functional group tolerance that included ethers (**58**), various halides (**59**–**60**), and nitro groups (**63**) (Scheme 6A). Interestingly, the reaction could also be applied to a sterically encumbered substrate bearing an *O*-*tert*-alkyl moiety, with the intended product (**62**) being isolated in 57% yield. The authors also proposed a mechanism to account for this reaction beginning with diaryliodonium salt (**I**) which upon exposure to TBAF leads to deprotection of the trisubstituted hydroxylamine to an *N*,*N*-disubstituted hydroxylamine (**II**) that undergoes intramolecular attack affording an iodaoxazepine (**III**) (Scheme 6B). Iodaoxazepine (**III**) can then undergo nitrogen attack at the *ipso*-carbon affording *N*-oxide (**V**) after the loss of iodobenzene through a proposed four-membered transition state (**IV**). Subsequent [1,2]-Meisenheimer rearrangement affords the intended products (**XI**) [54]. A radical pair pathway for the final step was supported by (2,2,6,6-tetramethylpiperidin-1-yl)oxyl (TEMPO) trapping studies, which led to the isolation of benzyl TEMPO adducts (**VIII**). For allylic substituted substrates that do not bear an *O*-benzyl group, the final step was proposed to proceed through a [2,3]-sigmatropic Meisenheimer rearrangement [54].

**Scheme 7:**
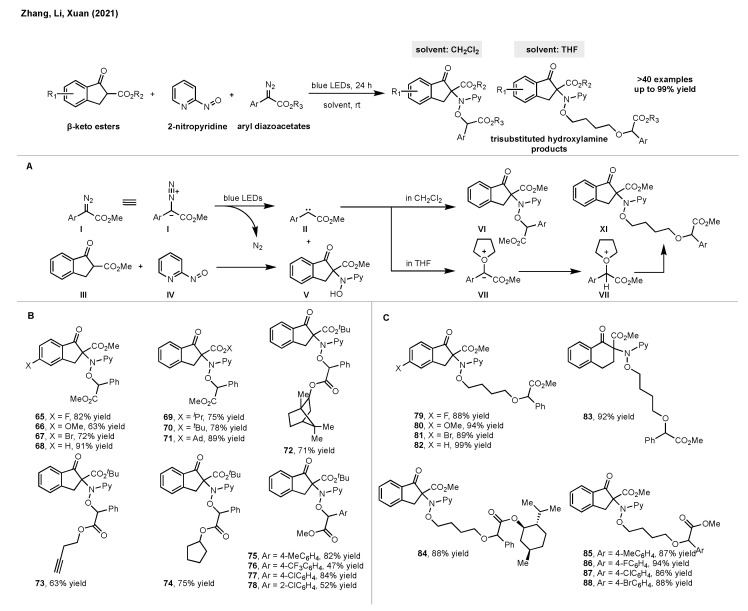
Zhang, Li, and Xuan’s multicomponent synthesis of trisubstituted hydroxylamines. (**A**) Selected examples of the transformation using CH_2_Cl_2_ as solvent. (**B**) Selected examples of the reaction using THF as solvent. (**C**) Proposed mechanism to account for differential reactivity in alternative solvents [55].

In 2021, Zhang, Li, and Xuan reported a blue-light-promoted multicomponent synthesis of trisubstituted hydroxylamines derived from 2-nitrosopyridine, aryl diazoacetates, and β-ketoesters (Scheme 7) [55]. Depending on the solvent, tetrahydrofuran (THF) or dichloromethane (CH_2_Cl_2_), trisubstituted hydroxylamines are formed either directly from the three substrates or with the insertion of a THF-derived butoxy chain. The authors rationalized this observation in terms of photolytic generation of a carbene (**II**) [56] that is then quenched by reaction with the adduct of the β-ketoesters (**III**) with 2-nitrosopyridine (**IV**) in CH_2_Cl_2_ to afford products (**VI**). However, in THF, the carbene species (**II**) first reacts with the solvent to afford an oxonium ylide (**VII**) that can undergo nucleophilic attack by the *N*,*O*-disubstituted hydroxylamine (**V**) to afford products (**XI**) [57]. Under both conditions, the reaction proceeds in excellent yield with very good functional group tolerance including various aryl halides (**65**, **66**, **79**, **81**) and complex alcohol-derived aryl diazoacetates (**72**–**78**, **84**–**88**), with hydroxylamines isolated in up to 99% yield (Scheme 7B,C).

**Scheme 8:**
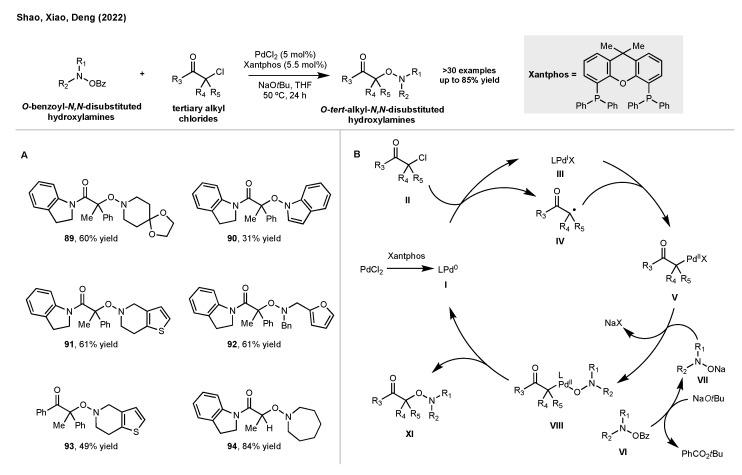
Shao, Xiao, and Deng’s synthesis of *O-tert*-alkyl-*N*,*N*-disubstituted hydroxylamines through palladium catalysis. (**A**) Selected examples of the transformation. (**B**) Proposed catalytic cycle [58].

In 2022, Shao, Xiao, and Deng reported a synthesis of sterically encumbered *O-tert*-alkyl-*N*,*N*-disubstituted hydroxylamines by tandem in situ deacetylation of *O*-benzoyl-*N*,*N*-disubstituted hydroxylamines and coupling with α-amido or α-keto tertiary alkyl chlorides (Scheme 8) [58]. The reaction proceeds under palladium catalysis with Xantphos as a ligand in up to 85% yield and exhibits good functional group tolerance. A wide variety of *N*-heterocycles were used in the reaction bearing diverse functionalities including dimethylketals (**89**), thiophenes (**91**), and furans (**92**) (Scheme 8A). The authors proposed a mechanism that begins with the reduction of Pd(II) to Pd (0) (**I**) followed by a single-electron transfer (SET) process between the Pd(0) and tertiary alkyl chloride, generating a Pd(I) species (**III**) and a tertiary alkyl radical (**IV**) (Scheme 8B) [59,60]. Subsequent recombination affords Pd(II) intermediate (**V**), which undergoes ligand exchange with in situ generated *N*,*N*-disubstituted hydroxylamine salt (**VII**) to afford alkyl-hydroxylamino Pd(II) intermediate (**VIII**). A final reductive elimination then furnishes the intended products (**XI**) and regenerates the Pd(0) catalyst (**I**).

**Scheme 9:**
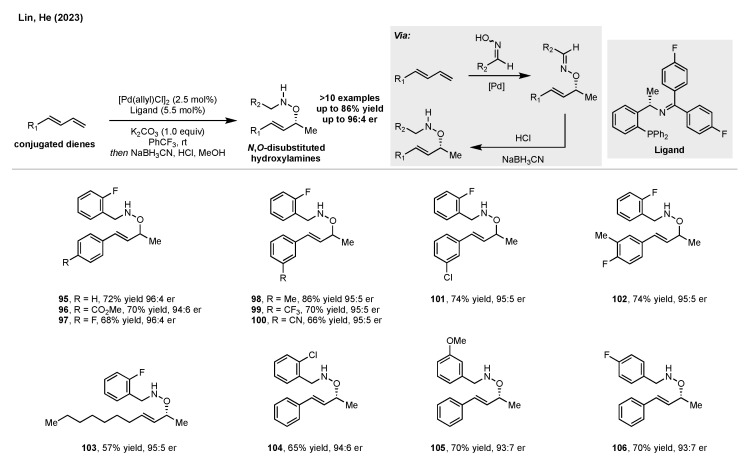
Lin and He’s catalytic asymmetric synthesis of secondary allylic hydroxylamines from conjugated dienes [61].

In 2023, Lin and He reported a novel catalytic asymmetric synthesis of allylic *N*,*O*-disubstituted hydroxylamines from conjugated dienes through a hydroaminoxylation procedure (Scheme 9) [61]. The reaction utilizes oxime nucleophiles under palladium catalysis to generate allylic oximes asymmetrically, which subsequently undergo reduction to the corresponding allylic *N*,*O*-disubstituted hydroxylamines. Under the optimized conditions, various functional groups were well tolerated in the transformation including methyl esters (**96**), aryl halides (**95**–**104**), nitrile (**100**), and trifluoromethyl (**99**) groups, affording products in good yields (68–74%) and excellent enantiomeric ratios (up to 96:4).

**Scheme 10:**
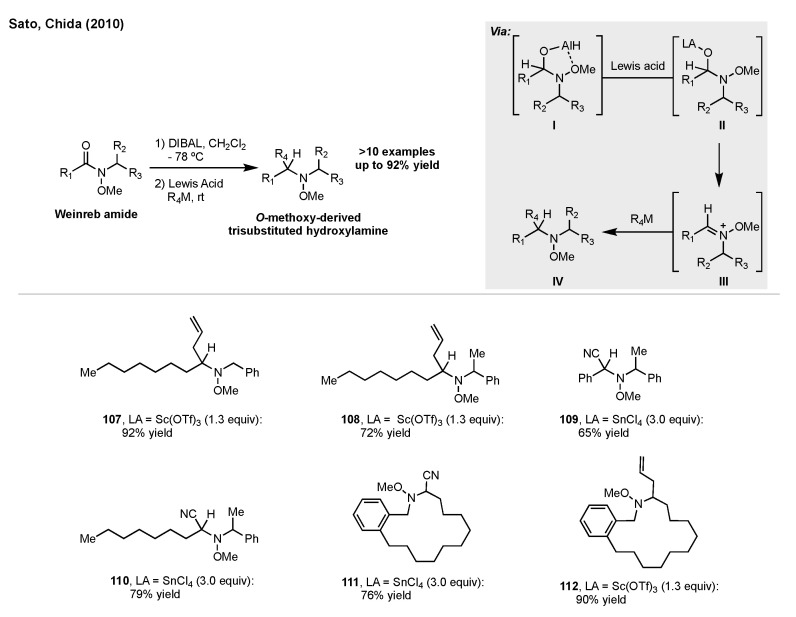
Sato and Chida’s α-functionalization of Weinreb amides for the synthesis of *O*-methoxy-derived trisubstituted hydroxylamines [62].

In 2010, Sato and Chida devised a method for the synthesis of *O*-methoxy-derived trisubstituted hydroxylamines with branching α- to the *N*-substituent [62]. The reaction consists of the reduction of Weinreb amides by diisobutylaluminum hydride (DIBAL) and subsequent trapping of the tetrahedral intermediate (**I**) with Lewis acids (**II**), which induces collapse to *N*-oxy-iminium ions (**III**) that can subsequently undergo attack with nucleophiles to afford products (**IV**) (Scheme 10). The reaction was tolerated by various alkyl substituents (**107**–**110**) and could also be applied in the synthesis of macrocyclic trisubstituted hydroxylamines giving products (**111**, **112**) in excellent yields (up to 90%).

**Scheme 11:**
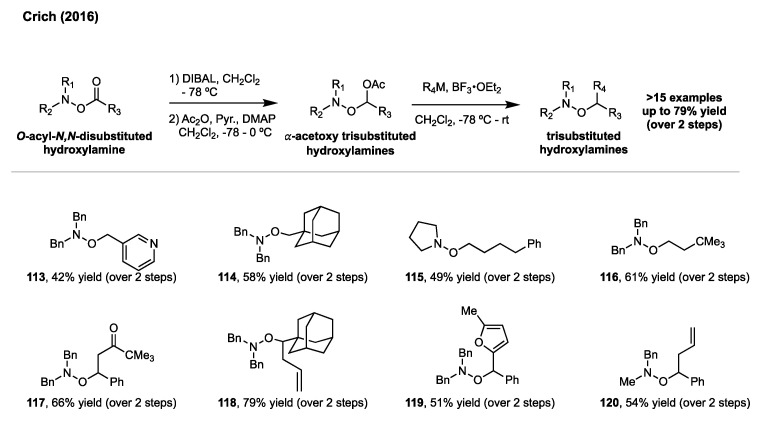
Crich’s *α*-functionalization of *O*-acyl-*N*,*N*-disubstituted hydroxylamines for the synthesis of trisubstituted hydroxylamines [63,64].

In 2016, Crich developed a method for the synthesis of trisubstituted hydroxylamines with branching α- to the *O*-substituent by application of Rychnovsky’s ether methodology to *O*-acyl-*N*,*N*-disubstituted hydroxylamines [63,64] (Scheme 11). Importantly, the reaction was not limited to *O*-methoxy derivatives as was the case in Sato and Chida’s reduction method [62]. Trisubstituted hydroxylamines could be readily synthesized with an α-methylene unit adjacent to the *O*-substituent as shown in trisubstituted hydroxylamine products (**113**–**116**), by reduction of the α-acetoxy intermediates with triethylsilane and BF_3_·OEt_2_. Moreover, trisubstituted hydroxylamines with alkyl branching α- to the *O*-substituent could be prepared using carbon-based nucleophiles in the last step, including silylenol ethers (**117**), 2-methylfurans (**119**), and allylmetals (**118**, **120**) all of which gave good to excellent yields of the hydroxylamine products (up to 79% yield, over two steps).

## 4. Synthesis of Hydroxylamines by [2,3]-Sigmatropic Rearrangements of *N*-Oxides and Cope-Type Hydroaminations

**Scheme 12:**
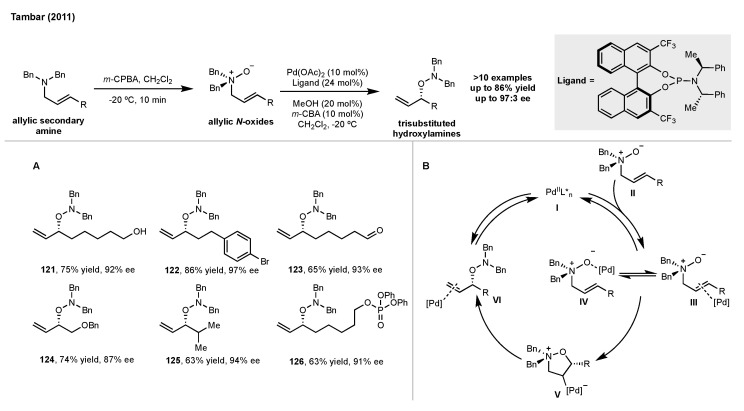
Tambar’s enantioselective [2,3]-sigmatropic rearrangement of allylic amine *N*-oxides for the synthesis of trisubstituted hydroxylamines. (**A**) Selected examples of the transformation. (**B**) Proposed catalytic cycle [67].

The [2,3]-Meisenheimer rearrangement, is a [2,3]-sigmatropic rearrangement of tertiary allylic *N*-oxides to trisubstituted hydroxylamines and represents a highly efficient, atom-economical route for the preparation of allylic trisubstituted hydroxylamines [65]. Recent work on [2,3]-Meisenheimer rearrangements has primarily focused on the development of asymmetric variants [66]. The first asymmetric Meisenheimer rearrangement was reported by Tambar in 2011 and was performed under palladium catalysis with an electron-deficient phosphoramidite ligand (Scheme 12) [67]. Interestingly, during optimization, the authors found that the addition of *meta*-chlorobenzoic acid (*m*-CBA) led to enhanced enantioselectivity in the reaction but did not provide an explanation for this phenomenon. Under the optimized conditions, a variety of allylic-dibenzylamines underwent efficient rearrangement after oxidation to the corresponding *N*-oxides in up to 86% yield with excellent enantioselectivity (up to 97:3) (Scheme 12A). The authors proposed a catalytic cycle whereby the Pd(II)-phosphoramidite catalyst (**I**) first acts as a π-acid and activates *N*-oxide (**II**) to an intermediate that exists either as an olefin-bound (**III**) or oxide-bound complex (**IV**) that leads to the formation of heterocycle (**V**) (Scheme 12B) [68]. The intermediacy of heterocycle (**V**) was supported by the lack of reactivity for substrates substituted at C-2 of the allylic functionality, which would be unable to form heterocycle (**V**) for steric reasons. A final aza-Grob fragmentation was proposed to afford the corresponding products (**VI**) and regenerate the Pd(II)-phosphoramidite catalyst (**I**) [69].

**Scheme 13:**
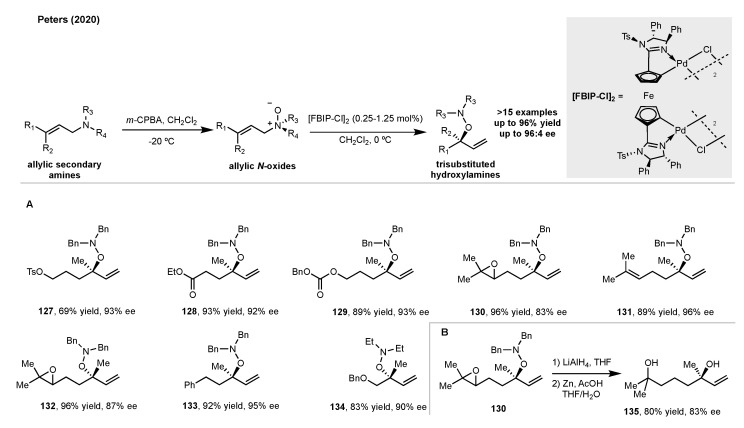
Peters’ enantioselective [2,3]-sigmatropic rearrangement of allylic amine *N*-oxides for the synthesis of *O*-*tert*-alkyl trisubstituted hydroxylamines. (**A**) Selected examples of the transformation. (**B**) Elaboration of the products to complex tertiary alcohols [67,70].

In 2020, Peters reported a method for the enantioselective synthesis of *O*-*tert*-alkyl allylic trisubstituted hydroxylamines under catalysis by a planar chiral ferrocene-based bispalladacycle, without the need for the exogeneous *m*-CBA needed in Tambar’s study (Scheme 13) [67,70]. The mild nature of these reaction conditions was underlined by the excellent functional group tolerance, which included substrates bearing primary tosylates (**127**), esters (**128**–**129**), and epoxides (**130**, **132**), from which all hydroxylamines were isolated in good to excellent yields (69–96%) and excellent enantioselectivity (up to 96:4) (Scheme 13A). The reaction could also be applied in the synthesis of sterically congested tertiary alcohols by reductive cleavage of the N-O bond as shown in the synthesis of **135**, which was accomplished in 80% yield over two steps (Scheme 13B).

**Scheme 14:**
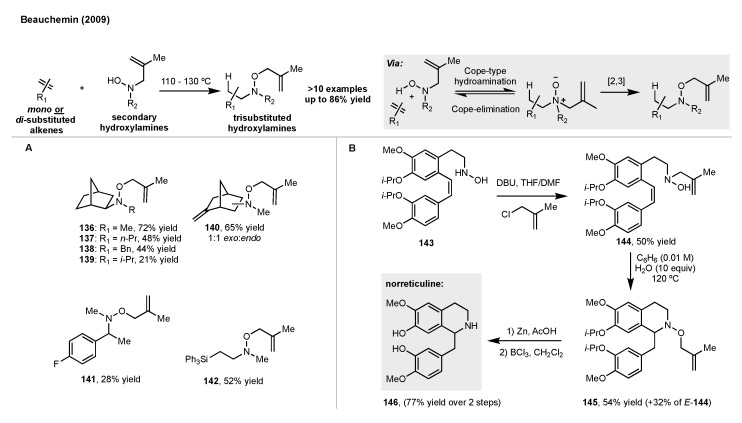
Beauchemin’s tandem Cope-type hydroamination/[2,3]-rearrangement for the synthesis of trisubstituted hydroxylamines. (**A**) Selected examples of the transformation. (**B**) Application of the method to the total synthesis of the alkaloid norreticuline [74].

The Cope elimination of tertiary *N*-oxides affords alkenes and hydroxylamines via a concerted, 5-membered cyclic transition state. In the reverse direction, the process is known as the Cope-type hydroamination [71,72,73]. This latter process has been used extensively, most notably by the Beauchemin group, as a highly efficient method for the preparation of *N*,*N*-disubstituted hydroxylamines and tertiary *N*-oxides [73,74,75,76,77,78,79,80]. In 2009, Beauchemin reported a synthesis of trisubstituted hydroxylamines by the reaction of mono or disubstituted alkenes with *N*,*N*-disubstituted hydroxylamines [74]. To overcome the propensity for reversible Cope-elimination of the Cope-type hydroamination *N*-oxide products, the authors appended a methallyl group on the hydroxylamine that undergoes an irreversible [2,3]-Meisenheimer rearrangement to afford the intended trisubstituted hydroxylamines (Scheme 14). The reaction was compatible with a variety of bicyclic alkenes (**136**–**140**) and amines (**141**–**142**), with products typically isolated in fair to good yields (up to 86%) (Scheme 14A). Beauchemin and coworkers also applied their methodology in the total synthesis of the alkaloid norreticuline (**146**) (Scheme 14B). Starting from *N*-monosubstituted hydroxylamine (**143**), installation of the methallyl moiety on **144** proceeded in 50% yield, which on exposure to heat underwent the desired tandem Cope-type hydroamination, [2,3]-Meisenheimer rearrangement, affording the allylic trisubstituted hydroxylamine (**145**) in 54% yield. Reductive cleavage of the N-O bond followed by BCl_3_-mediated cleavage of the *i*-Pr ethers yielded norreticuline (**146**) in 77% yield over two steps.

**Scheme 15:**
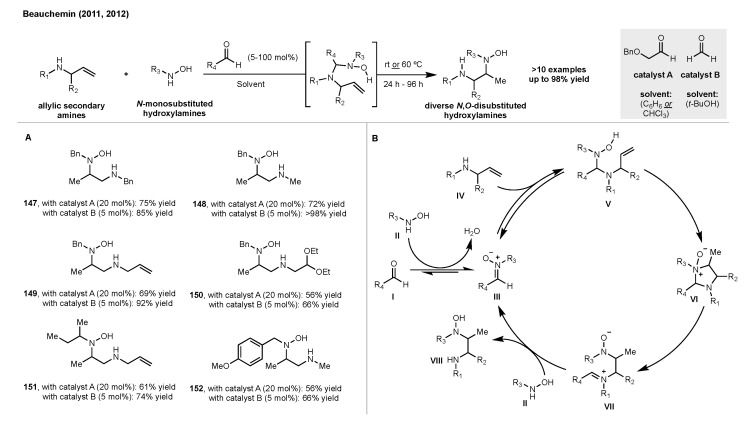
Beauchemin’s aldehyde catalyzed Cope-type hydroamination of alkenes for the synthesis of *N*,*O*-disubstituted hydroxylamines. (**A**) Selected examples of the reaction using different aldehyde catalysts. (**B**) Proposed catalytic cycle [75].

In a further strategy designed to overcome the reversible nature of Cope-type hydroaminations without recourse to a subsequent Meisenheimer rearrangement, in 2011 Beauchemin introduced a method based on “temporary intramolecularity” in which allylic secondary amines react with *N*-monosubstituted hydroxylamines in the presence of benzyloxyacetaldehyde (Scheme 15) [75]. In a subsequent 2012 study, Beauchemin showed that formaldehyde could act as a catalyst with improved yields at reduced loadings relative to that seen with the original benzyloxyacetaldehyde catalyst (Scheme 15A) [75,76]. Under both catalytic systems, a variety of functional groups were well tolerated including alkenes (**149**), diethylacetals (**150**), branched alkanes (**151**), and ethers (**152**) from which products were isolated in the highest yields under formaldehyde catalysis (up to 98% yield) (Scheme 15A). To account for the observed reactivity, the authors proposed a catalytic cycle beginning with an initial condensation of the aldehyde catalyst (**I**) and *N*-monosubstituted hydroxylamine (**II**), which affords an intermediate nitrone (**III**). The reaction of allyl amine (**IV**) in a carbon–nitrogen bond-forming event with **III** then affords mixed aminal (**V**), which undergoes the key intramolecular Cope-type hydroamination yielding *N*-oxide (**VI**). *N*-Oxide (**VI**) then undergoes fragmentation to zwitterionic species (**VII**), which upon reaction with another equivalent of hydroxylamine (**II**) regenerates nitrone catalyst (**III**) and furnishes the intended products (**VIII**).

## 5. Synthesis of Hydroxylamines by Direct N-O Bond Formation

**Scheme 16:**
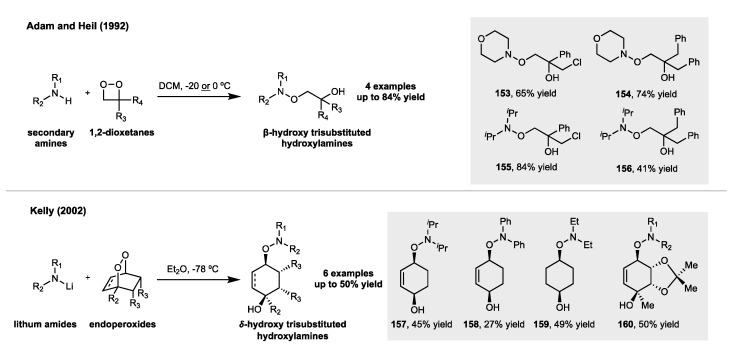
Early examples of direct N-O bond formation through reaction of amine nucleophiles with peroxide electrophiles by Adam, Heil, and Kelly and selected examples from the transformations [83].

Retrosynthetically, trisubstituted hydroxylamines could be accessed most efficiently by the attack of a secondary amine-based nucleophile on an oxygen-centered electrophile or by an alcohol-based nucleophile on an amine-centered electrophile. Unfortunately, such nucleophilic displacement reactions, termed X-philic reactions, are fraught with challenges due to competing eliminations and rearrangements [81]. Despite this, in nature, heteroatom-heteroatom bond formation is a common phenomenon and natural products-containing heteroatom-heteroatom bonds are found in many major classes of natural products [82]. Biosynthetically, the construction of hydroxylamines occurs by the reaction of amines with flavin-dependent *N*-monooxygenases (NMOs) or cytochrome P450 monooxygenase enzymes through enzyme bound peroxy-intermediates [82]. The use of peroxide O-O bonds as electrophilic “O^+^” sources in the biosynthesis of hydroxylamines is noteworthy as this method had not been developed in the laboratory for the preparation of hydroxylamines until recently on account of varied yields due to overoxidations [39]. Nevertheless, a few early examples of hydroxylamine synthesis by reaction of amine nucleophiles with specific classes of peroxide electrophiles have been reported in the literature. For example, in 1992 Adam and Heil reported the ring-opening reaction of 1,2-dioxetanes with amine nucleophiles for the synthesis of β-hydroxy trisubstituted hydroxylamines (**153**–**156**); yields of the transformation were generally good (up to 84% yield) (Scheme 16) [83]. Subsequently, in a 2002 mechanistic study of the Kornblum–DeLaMare rearrangement, the Kelly group reported the ring-opening reaction of *endo*-peroxides with lithium amides leading to δ-hydroxy trisubstituted hydroxylamines (**157**–**160**) (Scheme 16) [84]. Unfortunately, these early reports by Adam and Kelly have clear intrinsic scope limitations and as such, are not broadly applicable in the synthesis of a diverse array of trisubstituted hydroxylamines.

**Scheme 17:**
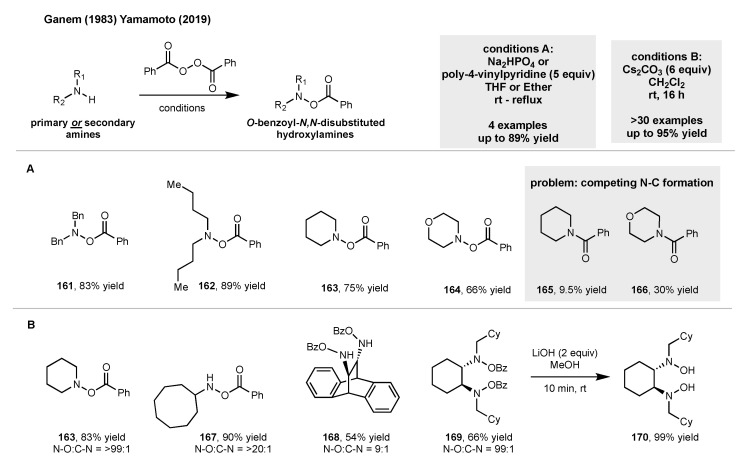
Ganem and Yamamoto’s syntheses of *O*-acyl-*N*,*N*-disubstituted hydroxylamines. (**A**) Selected examples and limitations of Ganem’s method (conditions A). (**B**) Selected scope and related derivatization of Yamamoto’s optimized method to reduce competing C-N bond formation (conditions B) [85,86].

An early example of a synthetic method that utilizes peroxide electrophiles and that has received broad usage in synthetic chemistry is the oxidation of amines with benzoyl peroxide (Scheme 17) [85]. Following the early reports, Ganem provided an improved procedure for the synthesis of *O*-benzoyl-*N*,*N*-disubstitued hydroxylamines that consists of an amine nucleophile reacting with benzoyl peroxide (BPO) as electrophile buffered by either Na_2_HPO_4_ or poly-4-vinylpyridine in ethereal solvents (Scheme 17). Under these conditions, sterically encumbered secondary amines such as dibenzylamine proceed smoothly to the corresponding *O*-benzoyl-*N*,*N*-disubstituted hydroxylamines (**161**–**164**) in up to 89% yield (Scheme 17A). However, with less sterically hindered secondary amines, competing N-C bond formation leading to the corresponding benzamides persists as a side reaction (**165**–**166**). Additionally, primary amines are unreactive substrates under Ganem’s original procedure. To overcome this issue, in 2019 Yamamoto published a revised experimental protocol that proceeds in excellent yields with high N-O selectivity (up to 99:1) (Scheme 17B) [86]. Under the optimized conditions, Cs_2_CO_3_ in CH_2_Cl_2_ with 3:1 BPO:water, a variety of primary amines and *N*-heterocycles such as piperidine and cyclooctanamine are well tolerated with the products (**163**, **167**) isolated in 83 and 90% yields, respectively (Scheme 17B). Additionally, under Yamamoto’s conditions, diamines can be used as substrates to yield *O*-benzoyl-*N*-monosubstituted hydroxylamine (**168**) in fair yield (54%) and good N-O selectivity (up to 9:1). Yamamoto also demonstrated deprotection of the *O*-benzoyl-*N*,*N*-disubstituted hydroxylamines to the corresponding *N*,*N*-disubstituted hydroxylamines by treatment with lithium hydroxide, where the intended product (**170**) was isolated in 99% yield.

**Scheme 18:**
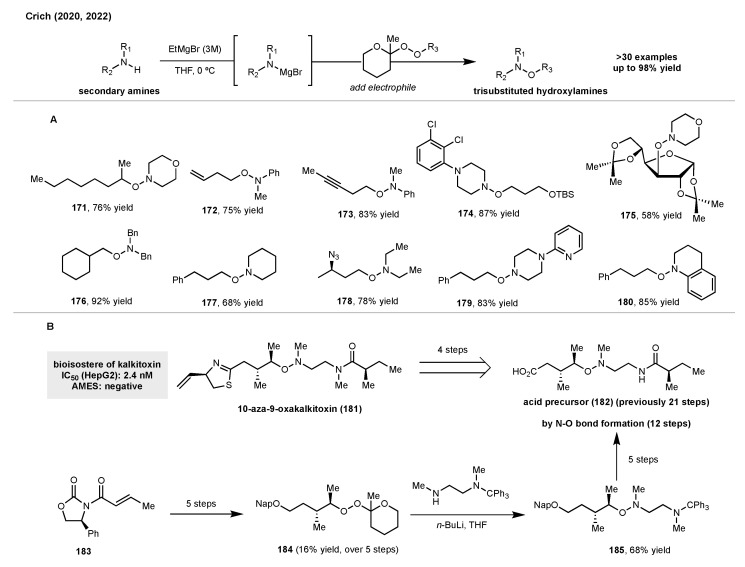
Crich’s synthesis of trisubstituted hydroxylamines by direct N-O bond formation. (**A**) Selected examples of the reaction. (**B**) Application of direct N-O bond formation to a shortened formal synthesis of a hydroxylamine analog of the marine natural product, kalkitoxin [87,92].

In 2020, Crich reported the first broadly applicable synthesis of trisubstituted hydroxylamines by direct N-O bond formation (Scheme 18) [87]. The method uses magnesium amides generated in situ as the nucleophilic component and alcohol-derived 2-methyl-tetrahydropyranyl (MTHP) monoperoxyacetals as electrophiles in an S_N_2-like reaction affording trisubstituted hydroxylamines in a direct, convergent manner [88,89,90]. A range of secondary amines in addition to primary and secondary alcohol-derived monoperoxyacetals bearing diverse functionalities were competent partners in the transformation. Compatible functional groups included internal alkenes (**172**), internal alkynes (**173**), aryl halides (**174**), complex carbohydrates (**175**), azides (**178**), or basic nitrogen heterocycles (**179**), all of which generally gave excellent yields (up to 98%) (Scheme 18A). Importantly, the reaction was also applicable in total synthesis as demonstrated by 10-aza-9-oxakalkitoxin (**182**), a hydroxylamine analog (hydroxalog) of the marine natural product kalkitoxin, that was synthesized in 16 steps using direct N-O bond formation as a key step [91], representing a marked improvement to Crich’s original 25 step synthesis of this molecule (Scheme 18B) [92].

**Scheme 19:**
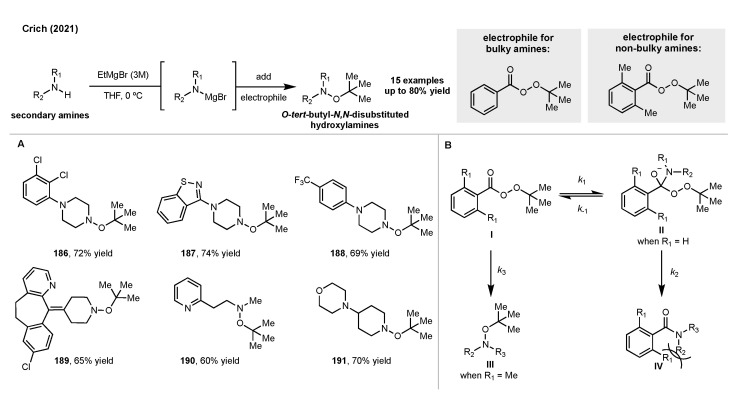
Crich’s synthesis of *O-tert*-butyl-*N*,*N*-disubstituted hydroxylamines by direct N-O bond formation using sterically matched electrophiles. (**A**) Selected examples of the reaction. (**B**) Proposed reaction pathway accounting for selective addition [87].

In the 2020 report [87], it was noted that *O-tert*-butyl-derived MTHP monoperoxyacetals failed under the optimized conditions to yield *O-tert*-butyl-*N*,*N*-disubstituted hydroxylamines (Scheme 18). In order to overcome this scope limitation, in 2021 Crich reported the synthesis of *O-tert*-butyl-*N*,*N*-disubstituted hydroxylamines by direct N-O bond formation using perester electrophiles (Scheme 19) [93,94]. Interestingly, the authors noted a steric requirement in the reaction whereby sterically encumbered magnesium amides reacted well with either *tert*-butylperbenzoate (TBPB) or 2,6-dimethyl-*tert*-butylperbenzoate, but less sterically hindered amine nucleophiles reacted predominately in a 1,2-fashion to afford benzamides with TBPB whereas they reacted chemoselectively to yield trisubstituted hydroxylamines with 2,6-dimethyl-*tert*-butylperbenzoate. Under the optimized conditions using the “sterically matched” electrophile, a variety of amine nucleophiles, containing diverse functional groups including aryl halides (**186**), isothiazoles (**187**), trifluoromethyls (**188**), or basic nitrogen heterocycles (**190**–**191**) could be used in the reaction in good yields (up to 80%) (Scheme 19A). The steric requirement for the transformation was accounted for by an irreversible attack on the peroxy oxygen bond in competition with reversible carbonyl addition (**II**) (Scheme 19B). The process of 1,2-addition to unsubstituted TBPB occurs where *k*_1_ > *k*_3_ and is proceeded by irreversible collapse to the benzamides by *k*_2_. Upon substitution of TBPB with the 2,6-dimethyl analog, a retardation in 1,2-addition is observed such that *k*_3_ > *k*_1_ and irreversible N-O bond formation predominates leading to the intended products (**III**) [95,96,97]. The predominant N-O bond formation seen with sterically encumbered amines was proposed to arise from collapse to the amide (*k*_2_) being sufficiently slowed by steric interactions in the product benzamides (**IV**) and in the corresponding transition state (**II**).

## 6. Conclusions

Di- and trisubstituted hydroxylamines have considerable unexplored potential in bioorganic and medicinal chemistry. This review has summarized key developments in the past 15 years which have made di- and trisubstituted hydroxylamines more readily available and should better position the community to fill this void.
